# Diversity of Active States in TMT Opsins

**DOI:** 10.1371/journal.pone.0141238

**Published:** 2015-10-22

**Authors:** Kazumi Sakai, Takahiro Yamashita, Yasushi Imamoto, Yoshinori Shichida

**Affiliations:** Department of Biophysics, Graduate School of Science, Kyoto University, Kyoto, Japan; Universitat Politècnica de Catalunya, SPAIN

## Abstract

Opn3/TMT opsins belong to one of the opsin groups with vertebrate visual and non-visual opsins, and are widely distributed in eyes, brains and other internal organs in various vertebrates and invertebrates. Vertebrate Opn3/TMT opsins are further classified into four groups on the basis of their amino acid identities. However, there is limited information about molecular properties of these groups, due to the difficulty in preparing the recombinant proteins. Here, we successfully expressed recombinant proteins of TMT1 and TMT2 opsins of medaka fish (*Oryzias latipes*) in cultured cells and characterized their molecular properties. Spectroscopic and biochemical studies demonstrated that TMT1 and TMT2 opsins functioned as blue light-sensitive Gi/Go-coupled receptors, but exhibited spectral properties and photo-convertibility of the active state different from each other. TMT1 opsin forms a visible light-absorbing active state containing all-*trans*-retinal, which can be photo-converted to 7-*cis*- and 9-*cis*-retinal states in addition to the original 11-*cis*-retinal state. In contrast, the active state of TMT2 opsin is a UV light-absorbing state having all-*trans*-retinal and does not photo-convert to any other state, including the original 11-*cis*-retinal state. Thus, TMT opsins are diversified so as to form a different type of active state, which may be responsible for their different functions.

## Introduction

**Fig 1:**
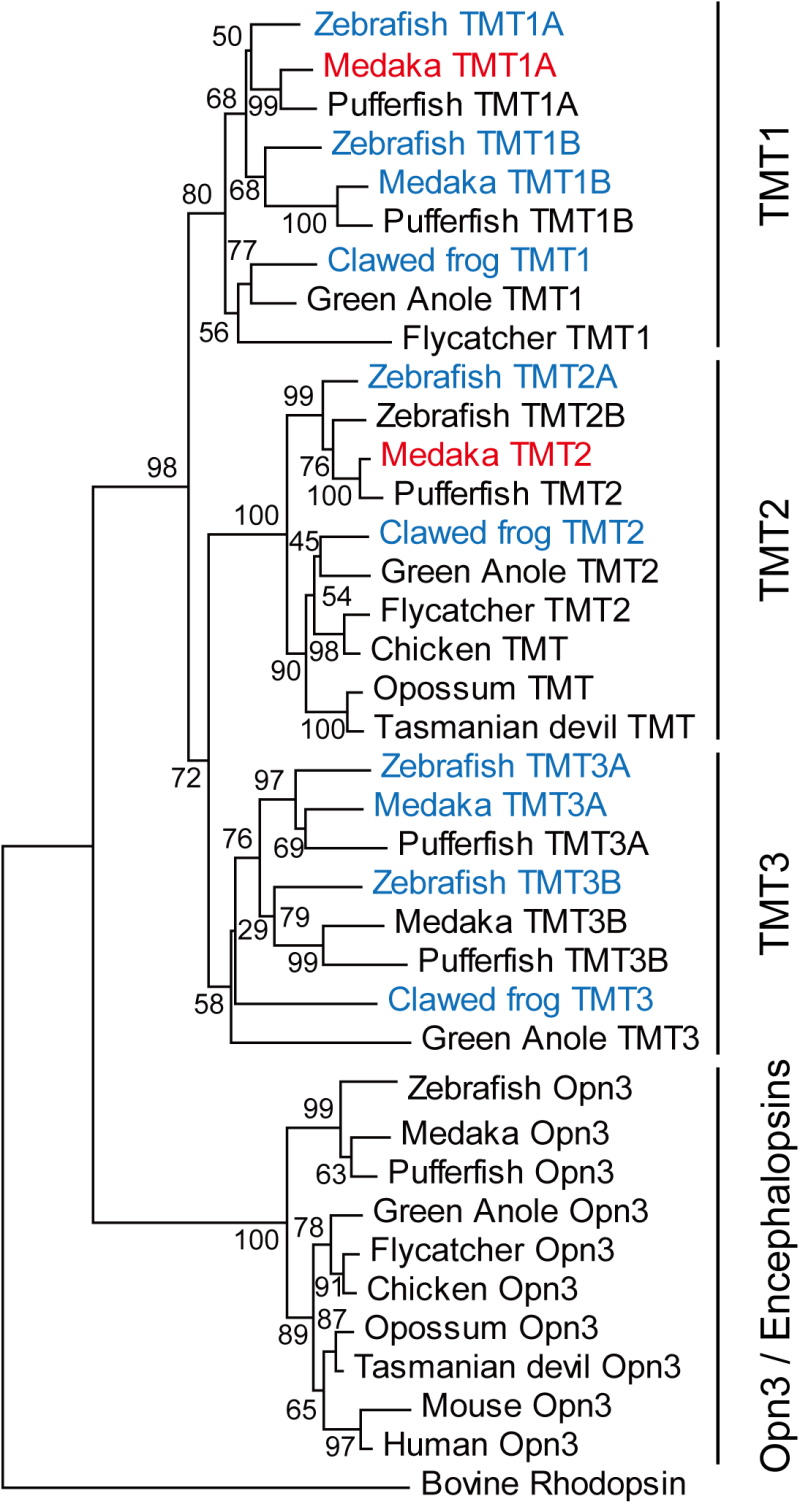
Phylogenetic analysis of vertebrate Opn3/TMTopsin group. The phylogenetic tree was constructed by the neighbor-joining method using MEGA 6 software [30]. Bootstrap probability (%) based on 1000 replicates is shown at the nodes. We tried to express TMT opsins from western clawed frog, zebrafish and medaka (blue letters) in cultured cells and successfully characterized the molecular properties of medaka TMT1A and TMT2 opsins (red letters). NCBI accession numbers used in this tree are as follows: zebrafish (BC163681, XM_002666663, NM_001281505, NM_001282373, NM_001282374, NM_001282375, EF043381), pufferfish (AF402774, XM_003968127, XM_003961790, XM_003970179, XM_003971042, XM_003963666), medaka (JX293354, JX293355, JX293356, JX293357, JX293358, NM_001305403), clawed frog (XM_002933372, LC009374, LC009375), green anole (XM_008106312, XM_008107065, XM_003228008, XM_008123887), flycatcher (XM_005050732, XM_005037520, XM_005043793), chicken (XM_004938518, XM_426139), opossum (JX293366, XM_001377887), Tasmanian devil (XM_003765692, XM_003767787), mouse (AF140241), human (AF140242) and bovine rhodopsin (NM_001014890).

Most animals have light-sensing G protein-coupled receptors called opsins to utilize light from the outer environment as various information sources. The agonist and inverse agonist of opsin are all-*trans*- and 11-*cis*-retinal, respectively. Opsins in the resting state contain inverse agonist 11-*cis*-retinal, which works as a light-absorbing chromophore. Light causes activation of opsin by changing 11-*cis*-retinal to agonist all-*trans*-retinal through photoisomerization. So far, several thousands of opsins have been identified from various animals, and have been classified into several groups based on their amino acid sequence identities [1, 2]. Opn3 (encephalopsin) was first identified in the deep brain and internal organs in human and mouse [3, 4]. After the discovery of mammalian Opn3, its homolog was found from not only neural tissues but also a variety of non-neural tissues of teleosts, and was named teleost multiple tissue opsin (TMT opsin) [5]. One of the characteristics of the Opn3/TMT opsin group is that, although it belongs to the vertebrate visual and non-visual opsin group, Opn3/TMT members have been isolated from both vertebrates and invertebrates [1, 6, 7]. Phylogenetic analysis of the Opn3/TMT opsin group showed that vertebrate Opn3/TMT opsin genes were divided into one Opn3 group and three distinct TMT opsin groups (TMT1, 2 and 3) (Fig 1) [8]. Opn3 is widely identified in vertebrates ranging from fish to mammals. TMT opsin is found from fish to birds and marsupials, but not in eutherians. Since TMT opsin is distributed in many tissues, including internal organs into which light is thought to scarcely penetrate in many vertebrates, TMT opsin might possibly have photoreceptive and non-photoreceptive functions. Recently, pufferfish (*Takifugu rubripes*) TMT1 opsin was found to be a photo-activated GPCR [6]; however, little is known about the detailed molecular properties of the members of this opsin group because of the difficulty of expressing the recombinant proteins.

To obtain insights into the physiological significance of the diversification of the Opn3/TMT opsin group, we have tried to express the recombinant proteins of opsins from this group to investigate their molecular properties. After several trials, we finally succeeded in preparing large enough amounts of the recombinant proteins of TMT1 and TMT2 opsins from medaka (*Oryzias latipes*) to use for photochemical and biochemical experiments. These two opsins exhibited absorption maxima at 460–470 nm and similar activation efficiencies of the Gi and Go subtypes of G protein. The active state of TMT1 opsin showed an absorption maximum in the visible region, and could be converted back to the original state together with other states containing 9-*cis*- and 7-*cis*-retinals by absorbing visible light. In contrast, the active state of TMT2 opsin exhibited an absorption maximum in the UV region, and was virtually “photo-insensitive”. We discuss the different molecular properties of TMT1 and TMT2 opsins in relation to the functional differences of these opsins.

## Materials and Methods

### Preparation of TMT opsin proteins

Four TMT opsin cDNA clones of medaka (GenBank accession numbers or NCBI reference sequence numbers: JX293354, JX293355, JX293356 and JX293357) were isolated by PCR from the 1st strand cDNA mixture of the brain. Five TMT opsin cDNA clones (BC163681, XM_002666663, NM_001281505, NM_001282374 and NM_001282375) of zebrafish (*Danio rerio*) were also isolated from the 1st strand cDNA mixture of the brain. We searched for the TMT opsin genes of western clawed frog (*Xenopus tropicalis*) in the NCBI database, and thus obtained the full-length ORF sequence of TMT1 opsin (XM_002933372) and isolated the cDNA clone from the 1st strand cDNA mixture of the brain. We also obtained the partial sequences of *Xenopus tropicalis* TMT2 (XM_002937662) and TMT3 (XM_002932623) opsins from the NCBI database. In the public genome database, the full-length ORF sequences of the two corresponding TMT opsins were predicted from the amino acid sequences of medaka and zebrafish clones. Using this information, we isolated full-length ORF cDNA clones of TMT2 (DDBJ accession number: LC009374) and TMT3 (LC009375) opsins from the 1st strand cDNA mixture of the brain of *Xenopus tropicalis*. Opsin cDNAs were tagged by the epitope sequence of anti-bovine rhodopsin monoclonal antibody rho1D4 (ETSQVAPA) at the C-terminus and were inserted into mammalian expression vector pMT4 [9] using the In-Fusion cloning technique (Clontech). To improve the expression or purification yield, we also constructed medaka TMT1A opsin cDNA whose C-terminal 63 amino acids were truncated.

The plasmid DNA was transfected into HEK293T cells by the calcium phosphate method. After 1 day of incubation, 11-*cis*-retinal was added to the medium (final concentration, 5 μM), and after additional incubation for 1 day in the dark, the cells were collected. The constituted pigments were extracted with 1% dodecyl maltoside (DM) in PM buffer (50 mM HEPES (pH 6.5), 140 mM NaCl, and 3 mM MgCl_2_) and were purified using a rho1D4-conjugated agarose column. The pigments were eluted with 0.02% DM in PM buffer containing the synthetic peptide that corresponds to the C-terminal sequence of bovine rhodopsin.

For experiments using the samples without detergents, membrane fragments expressing TMT opsins were prepared from HEK293T cells. Collected cells were suspended in 50% (w/v) sucrose in PM buffer (pH 7.0), sonicated and centrifuged. Then, the membrane fragments in the supernatants were precipitated by centrifugation after a 3-fold dilution with PM buffer. After washing three times with PM buffer, the membranes were used in the G protein activation assay. For spectroscopic measurements, the membranes were mixed with 20 mM hydroxylamine and incubated for 2 hours to remove the random Schiff base. After washing three times with PM buffer to remove residual hydroxylamine, the membranes were used for spectroscopic studies.

### UV−Visible spectroscopy and HPLC analysis

Absorption spectra were recorded using a UV-2400 spectrophotometer (Shimadzu). Opsin samples were irradiated with light from a 1 kW tungsten−halogen projector lamp which had passed through a glass cutoff filter and/or an interference filter.

The chromophore configurations were analyzed by HPLC (LC-10AT VP; Shimadzu) using a silica column (150 × 6 mm, A-012-3; YMC) according to a previous report [10].

### G protein activation assay

The rat Giα1 subunit was expressed in E. coli strain BL21 and was purified as described [11]. The purified Giα1 was mixed with an equal amount of purified bovine Gtβγ subunits [12]. Go-type of G protein was purified from pig cerebral cortex according to previous report [13]. A radionucleotide filter-binding assay, which measures GDP/GTPγS exchange by activated G protein, was performed as described previously [14]. All procedures were carried out at 0°C. The purified TMT opsins in PM buffer containing 0.02% DM, or TMT opsin-expressing membranes suspended in PM buffer, were mixed with G protein solutions and then pre-incubated for 1 hour at 0°C. For the measurements with TMT1A opsin, the samples were kept in the dark, and then irradiated with blue light (460 nm) for 40 sec, or irradiated with blue light for 40 sec and then with orange light (> 540 nm) for 5 min. For the measurement with TMT2 opsin, the samples were kept in the dark or irradiated with blue light (460 nm) for 1 min. After irradiation, the GDP/GTPγS exchange reaction was initiated by the addition of [^35^S]GTPγS to the mixture of the pigments and G proteins. The final assay mixture consisted of 50 mM HEPES (pH 7.0), 140 mM NaCl, 5 mM MgCl_2_, 1 mM DTT, 1μM GTPγS for Gi or 250 nM GTPγS for Go assay, 2 μM GDP for Gi or 5μM GDP for Go assay, 0.5 mg/ml L-α-phosphatidylcholine for Go assay, 500 nM G proteins and 50 nM pigments. After incubation for the indicated time in the dark, the reaction was terminated by adding stop solution (20 mM Tris/Cl (pH 7.4), 100 mM NaCl, 25 mM MgCl2, 1 μM GTPγS and 2 μM GDP), and it was immediately filtered through a nitrocellulose membrane to trap [^35^S]GTPγS bound to G proteins. The amount of [^35^S]GTPγS was quantitated by assaying the membrane with a liquid scintillation counter (Tri-Carb 2910 TR; PerkinElmer).

## Results

### Isolation of TMT opsin genes from vertebrates

As shown in Fig 1, vertebrate TMT opsins are divided into three groups. To characterize the molecular properties of TMT opsins, we isolated cDNA clones of TMT opsins from medaka, zebrafish and western clawed frog brains. However, we only succeeded in preparing recombinant proteins from medaka TMT1A and TMT2 opsins in Fig 1. Therefore, we described spectroscopic and biochemical properties of medaka TMT1A and TMT2 opsins in the following sections.

### Molecular properties of medaka TMT1A opsin

**Fig 2:**
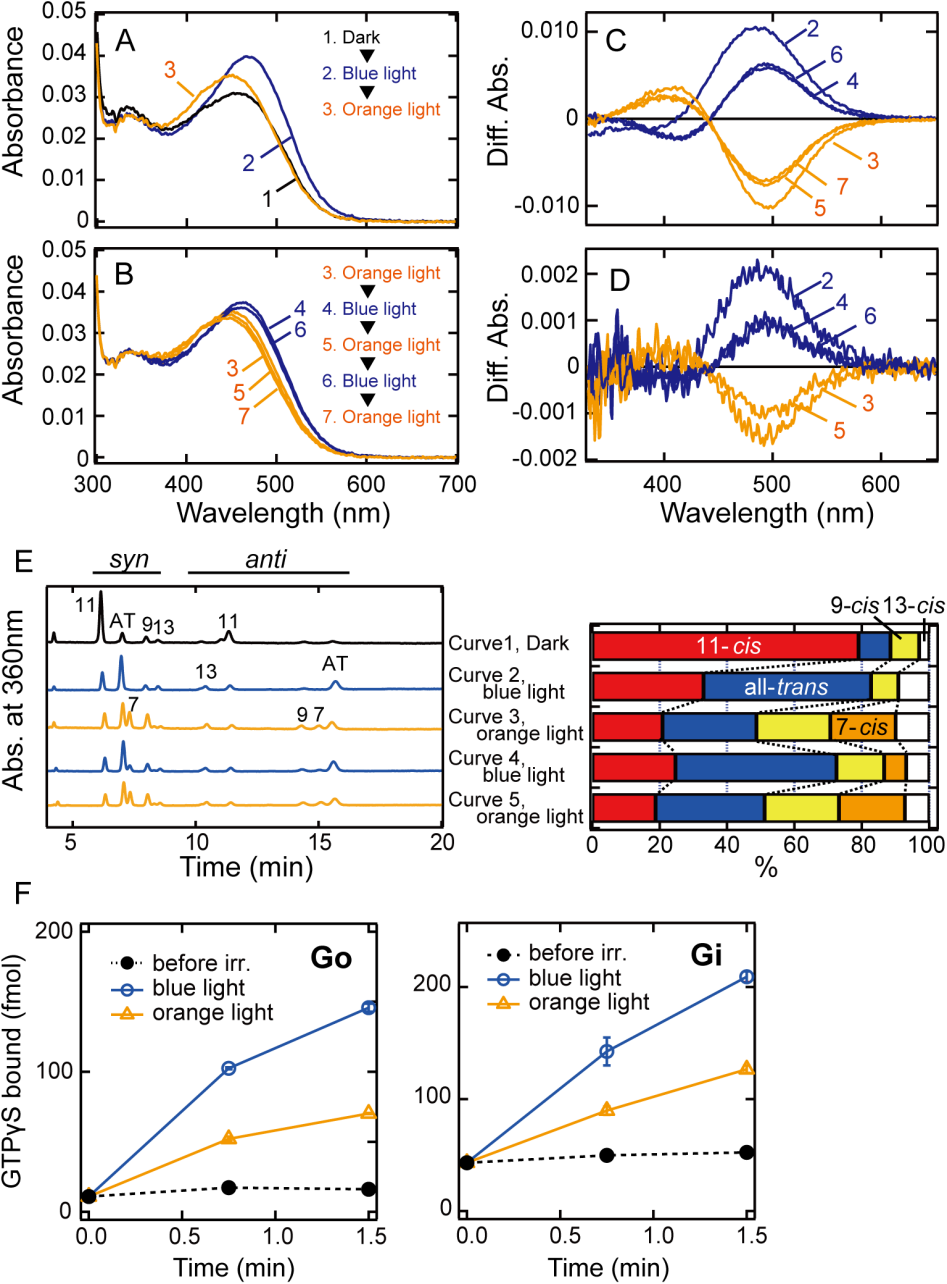
Absorption spectra and G protein activation abilities of medaka TMT1A opsin. (A) and (B) Photochemical reactions of TMT1A opsin at 0°C. TMT1A opsin sample (curve 1 in A) was irradiated with blue light (460 nm) for 40 sec (curve 2 in A), followed by irradiation with orange light (>540 nm) for 5 min (curves 3 in A and B). The sample was subsequently subjected to two more rounds of irradiation with blue and orange lights. That is, the sample was irradiated with blue light for 40 sec (curve 4 in B), with orange light for 5 min (curve 5 in B), with blue light for 40sec (curve 6 in B), and with orange light for 5 min (curve 7 in B). (C) Difference spectra calculated from the spectra shown in A and B. Curve 2 is the difference spectrum calculated by subtracting curve 1 in A from curve 2 in A. Curve 3 is that calculated by subtracting curve 2 in A from curve 3 in A. Curve 4 is that calculated by subtracting curve 3 in B from curve 4 in B. Curve 5 is that calculated by subtracting curve 4 in B from curve 5 in B. Curve 6 is that calculated by subtracting curve 5 in B from curve 6 in B. Curve 7 is that calculated by subtracting curve 6 in B from curve 7 in B. (D) Difference spectra calculated from the spectra recorded during experiments on photochemical reactions of TMT1A opsin in membrane fractions. Irradiation procedures performed during experiments on TMT1A opsin in membrane fractions, and the procedures for calculating difference spectra were the same as those described in A and B, and in C, respectively. (E) Analysis of retinal configurations of TMT1A opsin. Retinal composition changed after blue light irradiation, subsequent orange light irradiation, blue light re-irradiation, and orange light re-irradiation. Curve numbers indicate the absorption spectra shown in A and B. (F) Time courses of G protein activations by purified TMT1A opsin. The extents of Go and Gi activations were measured in the dark (closed circles), after blue light irradiation (open circles) and after subsequent orange light irradiation (open triangles). Experiments were performed using 50 nM pigment and 500 nM G proteins at 0°C. Data are presented as the means ± S.E.M. of three independent experiments.

Several previous reports showed that truncation of the C-terminal region of opsins could improve the expression or purification yield without significant alteration of their photochemical properties [6, 15]. Therefore, we prepared a truncated medaka TMT1A opsin construct in which 63 amino acid residues from the C-terminus were deleted, and found that the expression yield of the recombinant protein was about ten times larger than that of the full-length TMT1A opsin (S1 Fig). The absorption spectrum of purified TMT1A opsin exhibited an absorption maximum (λ_max_) at 460 nm (curve 1 in Fig 2A), which is similar to that of pufferfish TMT1 opsin previously reported [6]. Irradiation with blue light (460 nm) for 40 sec caused a slight red shift of λ_max_ and an increase in absorbance (curve 2 in Fig 2A). This indicated the formation of an intermediate having λ_max_ slightly longer than that of the original state. The wavelength of λ_max_ of the intermediate in the visible region strongly suggests that the intermediate would have a protonated Schiff base chromophore. HPLC analysis of the configurations of retinal chromophores extracted from the unirradiated and irradiated samples revealed that the conversion to the intermediate from the original state resulted from the cis-trans photoisomerization of the retinal chromophore (curves 1 and 2 in Fig 2E).

To investigate the photochemical properties of the all-trans product formed by irradiation with 460 nm light, we then irradiated the sample with orange (>540 nm) light for 5 min. This irradiation caused a shift of the λ_max_ to about 445 nm (curve 3 in Fig 2A), which is shorter than the λ_max_ (460 nm) of the original state (curve 1 in Fig 2A). Analysis of the chromophore configurations by HPLC showed the formation of products having 9-cis and 7-cis forms (Fig 2E) by this irradiation. In squid and octopus rhodopsins, acid metarhodopsin (all-trans form) shows light-dependent conversion mainly to the original rhodopsins (11-cis form) at 0°C [16, 17], whereas metamelanopsin (all-trans form) is converted to extramelanopsin (7-cis form) in addition to the original melanopsin (11-cis form) [10]. Thus, the all-trans product formed from TMT1A opsin has a tendency to easily convert to mono-cis products other than the 11-cis form. Re-irradiation of the sample with blue light for 40 sec caused a red shift of the spectrum (curve 4 in Fig 2B) due to the formation of all-trans and 11-cis products concurrently with the decrease of 9-cis and 7-cis products. Further irradiation with orange light for 5 min caused the formation of a state similar to that formed by the first 5-min irradiation with orange light (curve 5 in Fig 2B). We further observed cyclic changes of the spectra by alternating irradiations with blue and orange light (curves 4 to 7 in Fig 2B and 2C). These results strongly suggested that the all-trans product can photo-convert to the mono-cis products, including the original 11-cis form, and the mono-cis products can go back to the all-trans product by absorbing light (Fig 2E). It should be noted that we also observed similar photoreactions in membrane fractions (Fig 2D), suggesting that the easy conversion to the mono-cis products is one of the characteristics of TMT1A opsin.

We also examined the G protein activation ability of TMT1A opsin (Fig 2F). Blue light irradiation of TMT1A opsin increased the G protein activity (open circles), indicating that the all-trans product is a G protein-activating state. Subsequent irradiation with orange light caused a 50–60% loss of activity (open triangles), which is consistent with the decrease of the amount of all-trans product. Orange light irradiation caused the formation of 9-cis and 7-cis products, but the loss of G protein activity was correlated with the loss of the amount of all-trans product. Thus, 9-cis and 7-cis products may be inactive states for G protein. It should be noted that the Gi activation efficiency of TMT1A opsin was about 10 times lower than that of bovine rhodopsin while the Go activation efficiency of TMT1A opsin was almost the same as that of bovine rhodopsin (data not shown), which is consistent with the work previous published [6].

### Molecular properties of medaka TMT2 opsin

**Fig 3:**
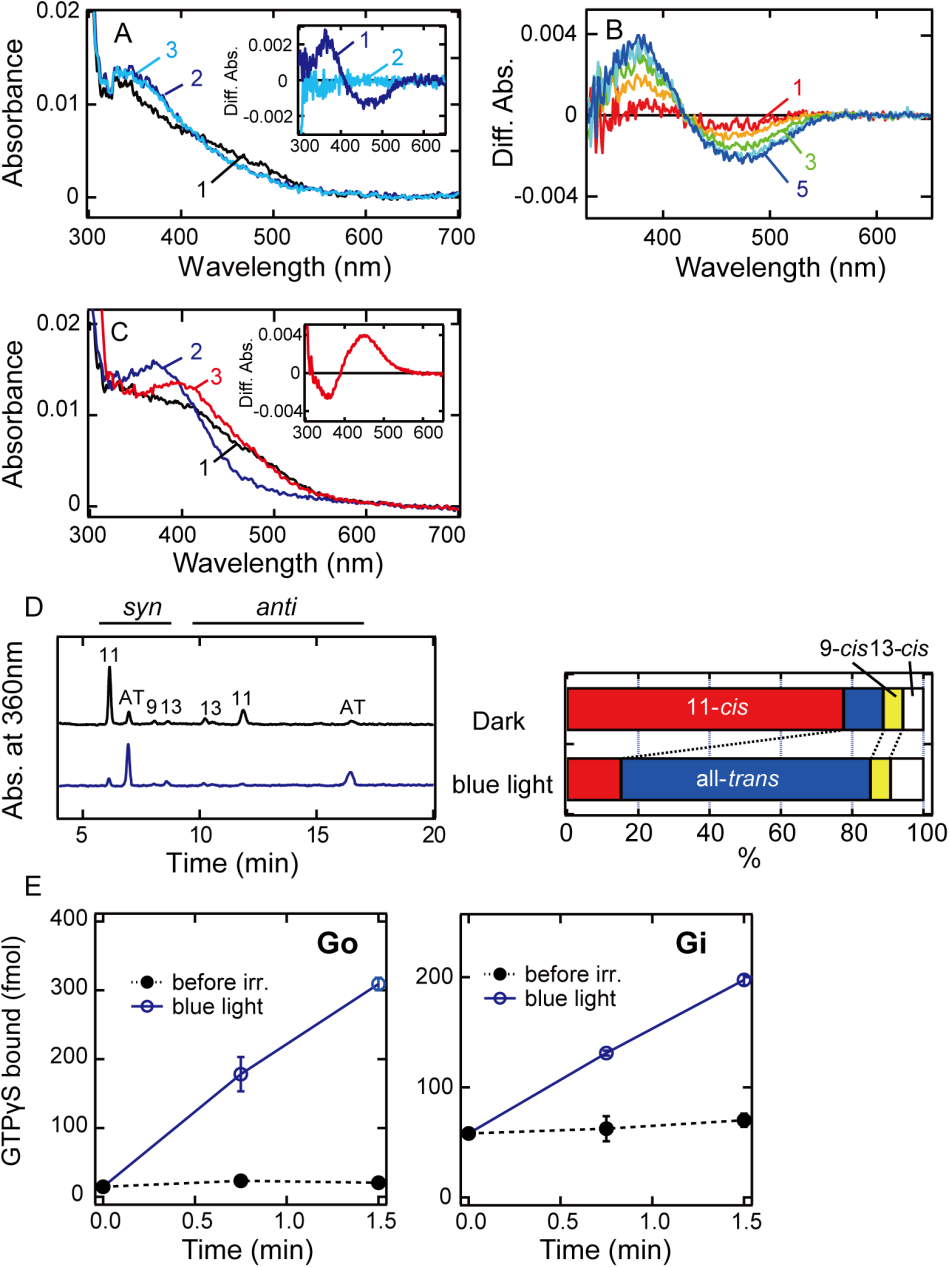
Absorption spectra and G protein activation abilities of medaka TMT2 opsin. (A) Absorption spectra of purified TMT2 opsin after reconstitution with 11-*cis*-retinal. Spectra were recorded before irradiation (curve 1), after blue light (460 nm) irradiation (curve 2) for 1 min and after subsequent UV light (360 nm) irradiation (curve 3) for 2 min at 0°C. (Inset) Spectral changes caused by blue light irradiation (curve 1) and subsequent UV light irradiation (curve 2). (B) Photoreactions of TMT2-expressing membrane fractions after reconstitution with 11-*cis*-retinal. The sample was successively irradiated with blue light for 10, 20, 40, 80, and 160 sec, and the difference spectra (curves 1–5) were calculated by subtracting the spectrum recorded before irradiation from those recorded after respective irradiations. (C) Absorption spectra before and after acid denaturation of TMT2 photoproduct. Spectra were recorded before irradiation (curve 1), after blue light irradiation (curve 2) and after subsequent addition of 2N HCl (curve 3). (Inset) The difference spectrum calculated by subtracting the spectra recorded before acidification from that recorded after acidification. (D) Analysis of retinal configurations of TMT2 opsin. Retinal composition changed after blue light irradiation. (E) Time courses of G protein activation abilities of purified TMT2 opsin. Go and Gi activation efficiencies were measured in the dark (closed circles) and after blue light irradiation (open circles). Experiments were performed using 50 nM pigments and 500 nM G proteins at 0°C. Data are presented as the means ± S.E.M. of three independent experiments.

We next investigated the photochemical and biochemical properties of TMT2 opsin. Because of the low expression yield of the recombinant protein, we only obtained a small amount of partially purified pigment of TMT2 opsin after reconstitution with 11-*cis*-retinal (curve 1 in Fig 3A). To improve the expression yield, we truncated twelve amino acid residues from the C-terminus of TMT2 opsin, but found no improvement in yield. Therefore, we used the full-length TMT2 opsin as our experimental sample. Blue light (460 nm) irradiation of the pigment resulted in a shift of the spectrum to the UV region (curve 2 in Fig 3A). The difference spectrum calculated by subtracting the spectrum recorded before blue light irradiation from that recorded after blue light irradiation exhibited a negative peak at about 470 nm (inset of Fig 3A). This should be a λ_max_ of the original dark state of TMT2 opsin, which is slightly longer than that of TMT1A opsin (λ_max_ = 460 nm). HPLC analysis showed that this photoreaction was accompanied by the cis-trans isomerization of the retinal chromophore (Fig 3D). To investigate the photo-convertibility of the all-trans product, we irradiated the sample with UV light (360 nm). However, the spectrum did not change (curve 3 in Fig 3A). To verify whether the absorbance of the photoproduct in the UV region was derived from an unprotonated intermediate or a retinal released from the protein, we performed an acid denaturation experiment (Fig 3C). That is, we expected that, if the sample contained a UV light-absorbing unprotonated intermediate, addition of HCl solution to the sample would denature the intermediate and trap the chromophore as a protonated Schiff base with λ_max_ of about 440 nm, whereas no spectral change would be observed if the retinal chromophore were already released from the protein. The results showed that acidification caused a red shift of the spectrum (curve 3 in Fig 3C). The difference spectrum calculated by subtracting the spectrum recorded before acidification from that recorded after acidification exhibited a positive peak at about 440 nm (inset of Fig 3C), which is indicative of the formation of the protonated Schiff base. These results showed that TMT2 opsin photo-converts to the unprotonated intermediate that does not photo-convert back to the original dark state. It should be noted that the addition of hydroxylamine to the TMT2 opsin sample caused no detectable spectral changes, suggesting that the absorbances in the near-UV region in the sample are due to contaminants other than the random Schiff base (S3 Fig). We also irradiated the TMT2 opsin-expressing membrane fractions and confirmed the occurrence of spectral changes similar to those in detergent solution (Fig 3B). Thus, we concluded that TMT2 opsin forms an unprotonated intermediate after light irradiation, which is apparently photo-insensitive.

Next, we measured Gi and Go activation abilities of purified samples of TMT2 opsin (Fig 3E). Blue light irradiation caused elevation of the amount of GTPγS binding to Gi and Go (open circles), indicating that the all-trans product was the G protein-activating state of TMT2 opsin.

## Discussion

In the present study, we characterized the molecular properties of medaka TMT1A and TMT2 opsins and found that they are both blue light-sensitive Gi/Go-coupled receptors, but form active states having different spectral and photochemical properties. TMT1A opsin photo-converts to an active state that has λ_max_ in the visible region and is photo-convertible to the original state (Fig 2E). This photo-reversibility is consistent with the spectral changes of pufferfish TMT1 opsin [6]. Therefore, TMT1 opsin could be categorized as a “bistable opsin” [18–20], with characteristics similar to those originally found in squid rhodopsin [16, 17]. However, unlike squid rhodopsin, TMT1A opsin forms an active state (all-trans state) that can easily convert to mono-cis states other than the original 11-cis state (Fig 2E). Since 7-cis and 9-cis states have λ_max_ at wavelength shorter than that of the original state, these characteristics might be advantageous for TMT1A opsin under bright light conditions, where TMT1A opsin converts to a mixture containing the active state, original 11-cis state, and other mono-cis states, and thus acquires the ability to respond to shorter wavelength light. The role of the mono-cis isomers other than the original 11-cis isomer in spectral extension was originally suggested from experiments on mouse melanopsin [10, 21]. Therefore, easy conversion to the mono-cis states other than the original 11-cis state from the active state might be one of the characteristics of non-visual opsins as compared to visual opsins such as bovine rhodopsin and squid rhodopsin. TMT1A opsin forms a mixture of active and inactive states under continuous light conditions, suggesting that it might modulate its response to a light signal by changing the ratio between the active and inactive states in response to the change of wavelength components in the external light environment, such as during dawn, daytime and dusk. It was reported that TMT1A opsin can trigger a light input pathway to the peripheral clock in peripheral tissues such as fin cells of teleosts [22]. The characteristic molecular properties of TMT1A opsin demonstrated here may contribute to time-dependent input to the peripheral clock. To elucidate the physiological significance, it is important to determine the precise absorption characteristics of TMT1A opsin and its photoproducts, which will be the focus of our future work.

In contrast, TMT2 opsin photo-converts to an active state that has λ_max_ in the UV region (Fig 3A). This is similar to the formation of meta II in bovine rhodopsin. However, the photoreaction of the UV light-absorbing active state is different between bovine rhodopsin and TMT2 opsin. Meta II was converted to meta III by UV light irradiation [23], whereas UV light irradiation of the active state of TMT2 opsin did not cause conversion to any other states (curve 3 in Fig 3A), which means that the active state of TMT2 opsin is virtually “photo-insensitive”. Therefore, TMT2 opsin can work under light conditions different from those that activate TMT1A opsin. That is, TMT2 opsin can accumulate the active state under weak light conditions, and can completely convert to the active state under bright light conditions. In fact, medaka TMT2 opsin is reported to be distributed exclusively in eyes and brain [8]. Thus, TMT2 opsin may function as a photoreceptor with high signal-to-noise ratio within the tissues where retinal supply is plentiful. It is reported that RPE65, an essential enzyme in the visual cycle, is expressed in the zebrafish brain [24]. Therefore, 11-*cis*-retinal could potentially be supplied to TMT opsins. Further analysis of the distribution patterns of the enzymes of the visual cycle will reveal a functional model for TMT opsins in the medaka brain.

TMT2 opsin is similar to vertebrate rhodopsin from the viewpoint of the formation of a UV light-absorbing active state. However, the mechanism of formation of the active state in TMT2 opsin is not clear yet. In vertebrate rhodopsin, retinal chromophore forms a protonated Schiff base with an amino group of a lysine residue, and the proton on the Schiff base is stabilized by a counterion, a negatively charged glutamic acid at position 113 [25, 26]. Light absorption causes cis-trans isomerization of the retinal chromophore, followed by proton transfer from the Schiff base to the counterion, resulting in rearrangements of the helices, especially that of helix VI, due to disappearance of the ion-lock structure between helices III and VII. In contrast, TMT2 opsin does not have a counterion at position 113, where it has a tyrosine residue instead of glutamic acid. Thus in TMT2 opsin, the proton on the Schiff base should be stabilized by glutamic acid at position 181 through hydrogen bonding networks including water molecules [27], and deprotonation of the Schiff base would occur in the process of formation of the active state. However, the active states of TMT1 and TMT2 opsins exhibited equivalent activation efficiency. These facts suggest that deprotonation of the Schiff base in TMT2 opsin would induce conformational changes in the protein moiety different from that in bovine rhodopsin, which would be one of the mechanisms leading to differences in the efficiency of G protein activation between TMT2 opsin and bovine rhodopsin.

To obtain more insights into the deprotonation mechanism in TMT2 opsin, we have tried to identify amino acid residue(s) responsible for the difference in the process of formation of the active state between TMT1 and TMT2 opsins. Based on the 3D structures of bovine and squid rhodopsins [28, 29], we compared amino acid residues located within 4.5 Å of the retinal chromophore between medaka TMT1A and TMT2 opsins and found that, unexpectedly, all the amino acid residues (total of 20 amino acids) are completely conserved. Then we searched for the amino acid residues located within 6 Å of the nitrogen atom of the Schiff base (14 amino acids) and found that there are 3 different amino acid residues between medaka TMT1A and TMT2. Among these residues, the amino acid residue at position 186 (bovine rhodopsin numbering) in the second extracellular loop was completely conserved in the respective groups (threonine in TMT1 opsin group and serine in TMT2 opsin group). We then constructed a single mutant (T186S) of TMT1A opsin and a chimeric mutant of TMT1A opsin whose second extracellular loop was replaced by that of TMT2 opsin (TMT1A-EL2-TMT2). However, these replacements did not change the photoreaction (S2 Fig). Therefore, we speculate that the diversity of the mechanism of formation of the active state in TMT opsins should be derived from amino acid residue(s) far from the retinal chromophore, suggesting that the difference should be transmitted to the retinal chromophore through whole protein dynamics. It should be noted that TMT2 opsin is the only opsin that has a UV light-absorbing active state among the opsins having a counterion at position 181. Therefore, elucidation of the deprotonation mechanism of TMT2 opsin could have some implications about the molecular evolution of vertebrate rhodopsin.

In summary, we found that TMT1 and TMT2 opsins have absorption spectra similar to each other, but photo-convert to active states with different characteristics. Although medaka TMT1 and TMT2 opsins are both expressed in eyes and brain, their distributions are somewhat different [8]. Therefore, the relationship between the different molecular properties and physiological functions of TMT1 and TMT2 opsins will be an important issue to be elucidated in the future.

## Supporting Information

S1 Fig Absorption spectrum of purified full-length TMT1A opsin. Full-length pigment was formed after reconstitution with 11-*cis*-retinal.
(TIF)

S2 Fig Photoreactions of TMT1A opsin mutants. (A) Amino acid sequences of the second extracellular loop of TMT1A and TMT2 opsins. (B, C) Absorption spectra of TMT1A-T186S (B) and TMT1A-EL2-TMT2 (C). Spectra were recorded before irradiation (black curve) and after blue light (460 nm) irradiation (red curve) for 1 min at 0°C. (Inset) Spectral changes caused by blue light irradiation.
(TIF)

S3 Fig Hydroxylamine sensitivity of TMT2 opsin. TMT2 opsin (curve 1) was incubated at 0°C in the presence of 20 mM hydroxylamine. Spectral change was recorded at 60 min (curve 2) after the addition of hydroxylamine. Then, the sample was irradiated with blue light (460 nm) for 2 min (curve 3).
(TIF)
