# Supplementary figures and images for: Diversity of Active States in TMT Opsins
Source: PLoS One. 2015 Oct 22;10(10):e0141238. doi: 10.1371/journal.pone.0141238 (PMC4619619; doi:10.1371/journal.pone.0141238)

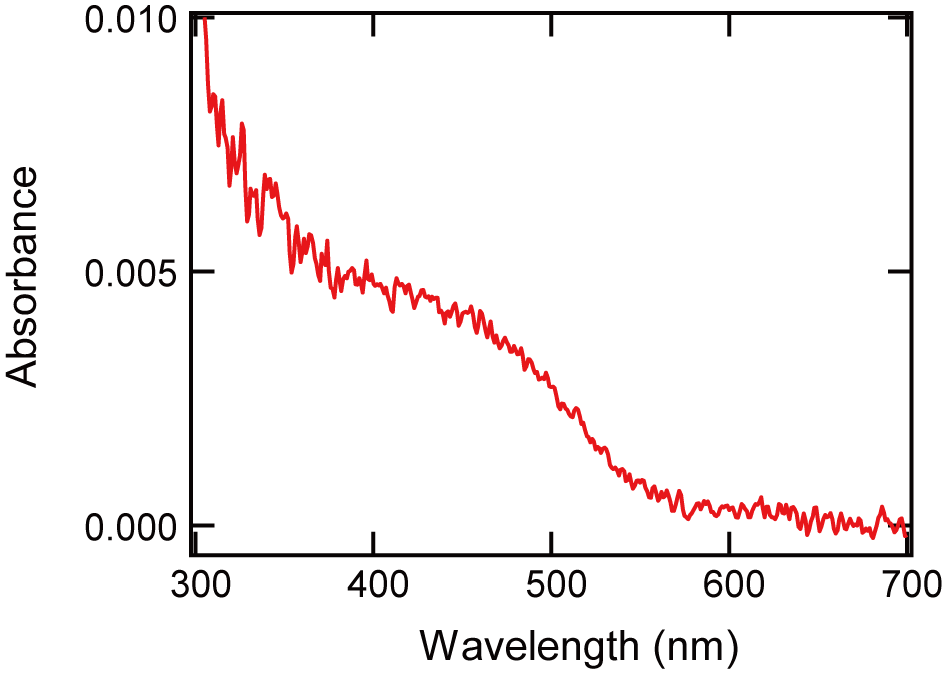

Supplement: S1 Fig — Full-length pigment was formed after reconstitution with 11-cis-retinal. (TIF) [file pone.0141238.s001.tif]

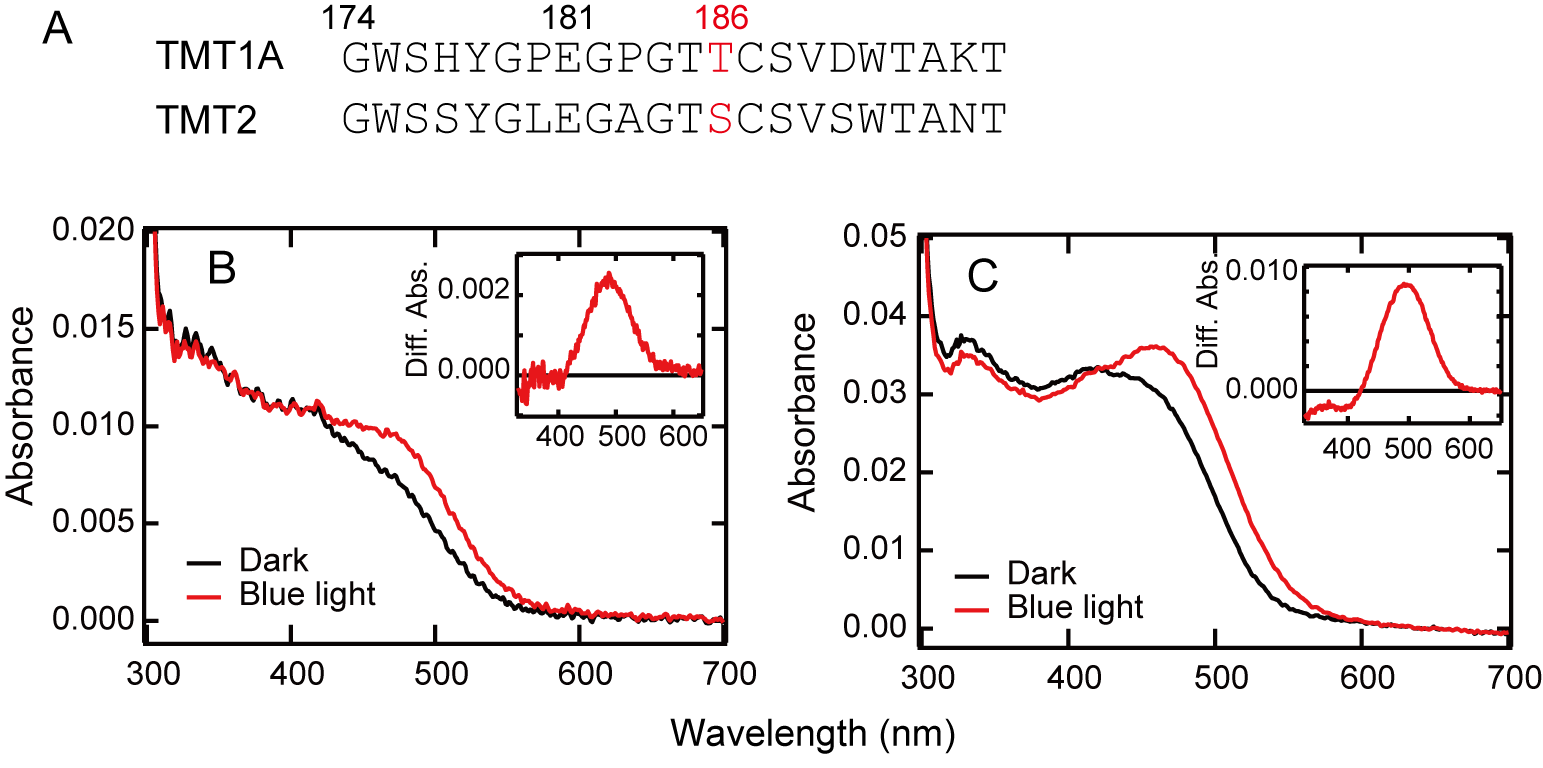

Supplement: S2 Fig — (A) Amino acid sequences of the second extracellular loop of TMT1A and TMT2 opsins. (B, C) Absorption spectra of TMT1A-T186S (B) and TMT1A-EL2-TMT2 (C). Spectra were recorded before irradiation (black curve) and after blue light (460 nm) irradiation (red curve) for 1 min at 0°C. (Inset) Spectral changes caused by blue light irradiation. (TIF) [file pone.0141238.s002.tif]

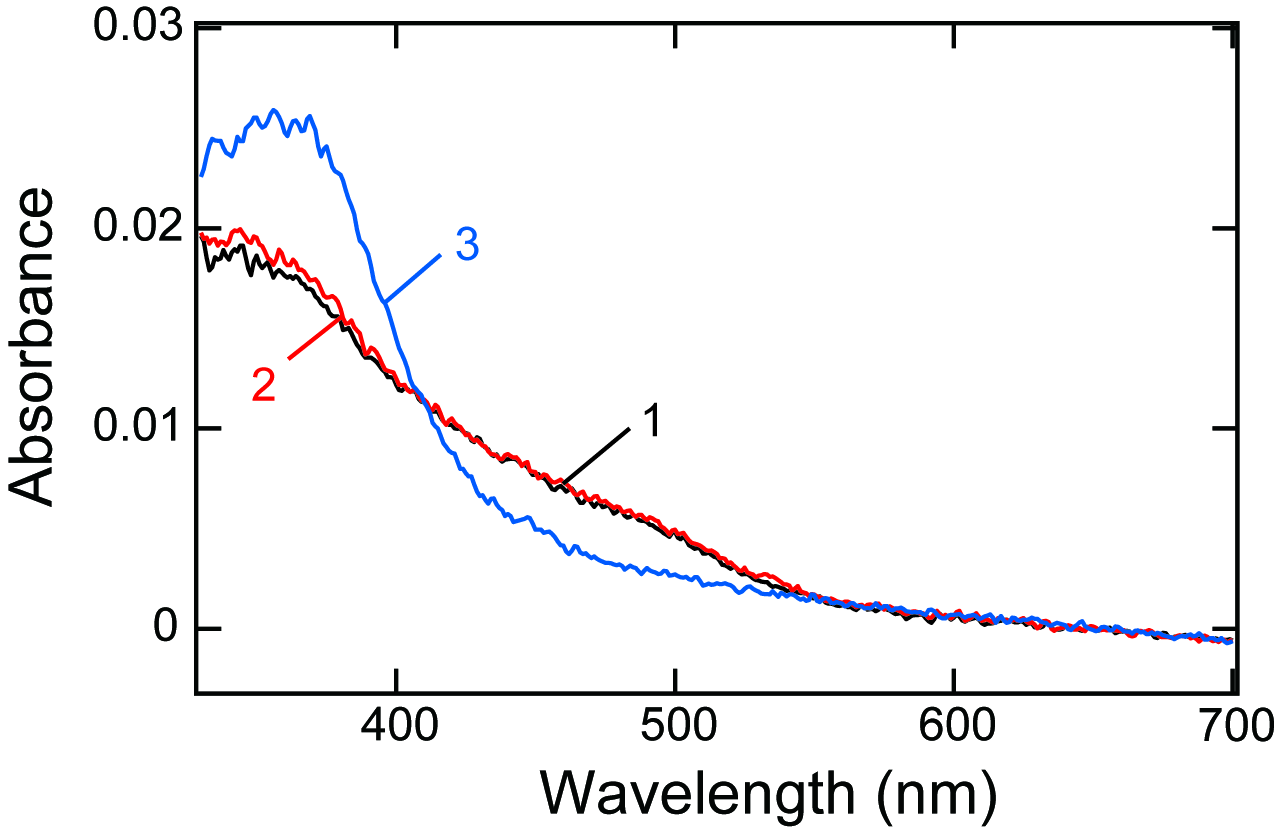

Supplement: S3 Fig — TMT2 opsin (curve 1) was incubated at 0°C in the presence of 20 mM hydroxylamine. Spectral change was recorded at 60 min (curve 2) after the addition of hydroxylamine. Then, the sample was irradiated with blue light (460 nm) for 2 min (curve 3). (TIF) [file pone.0141238.s003.tif]
